# Stratification of Hepatocellular Carcinoma Using *N*^6^-Methyladenosine

**DOI:** 10.3390/cancers17132220

**Published:** 2025-07-02

**Authors:** Nan Wang, Jia-Xin Shi, Matthias Bartneck, Edgar Dahl, Junqing Wang

**Affiliations:** 1 Department of General Surgery, Ruijin Hospital, Shanghai Jiao Tong University School of Medicine, Shanghai 200025, China; 2 Department of Internal Medicine III, University Hospital RWTH Aachen, 52074 Aachen, Germany; 3 Institute of Pathology, Medical Faculty, RWTH Aachen University, 52074 Aachen, Germany; 4 Center for Integrated Oncology Aachen Bonn Cologne Düsseldorf (CIO ABCD), 52074 Aachen, Germany

**Keywords:** hepatocellular carcinoma, m^6^A, survival prognosis, immunotherapy, ANLN

## Abstract

**Background**: The *N*^6^-methyladenosine (m^6^A) modification of eukaryotic mRNA is the most prevalent of such epigenetic modifications and has recently been identified as a potential player in the pathogenesis and progression of hepatocellular carcinoma (HCC). With the increasing emergence of immunotherapy in the treatment of HCC, we have evaluated the potential of m^6^A-related genes in predicting overall survival and the therapeutic efficacy of immunotherapy in HCC patients. **Methods**: We employed transcriptomic data from TCGA-LIHC and GSE76427, comprising a total of 485 HCC patients, as the training set. Based on 23 recognized m^6^A regulators, we performed clustering analysis on HCC patients. The intersecting differentially expressed genes (DEGs) among subtypes were used in least absolute shrinkage and selection operator (LASSO) Cox and multivariate Cox regression analyses to construct the risk model. For the quantification of a risk model of HCC patients, a risk score was developed and correlated with clinical and immunological parameters. Furthermore, a single-cell transcriptomic atlas was used to analyze the relationship between model genes and immune cell subpopulations. Mechanistic studies included in vitro assays to validate the association between the m^6^A-related gene *ANLN* and the progression of HCC. **Results**: Internal (TCGA and GEO) and external validation (ICGC) suggested that an 8-gene risk score provides an accurate and stable prognostic assessment for HCC. Furthermore, the high-risk score, characterized by elevated TP53 mutation frequency, tumor mutation burden (TMB), and tumor stem cell characteristics indicated a poor prognosis. The prognostic signature was associated with immune cell infiltration in HCC. Those patients with a high-risk score had lower immune tolerance with a better prediction of the efficacy of immunotherapy. The risk model helps to assess and predict the response and prognosis of HCC patients to immune checkpoint inhibitors (ICIs). Additionally, single-cell RNA sequencing data revealed that the high-risk group had a higher proportion of T cells and fewer immunosuppressive T cells, potentially correlating with a better response to immunotherapy. Finally, in vitro experiments showed that *ANLN*, an m^6^A-related gene, promoted the proliferation and migration of HCC cells. **Conclusions**: In this study, we identified and validated an m^6^A gene signature consisting of eight genes that can be used to predict prognosis and immunotherapy efficacy in HCC patients.

## Simple Summary

Hepatocellular carcinoma (HCC) is a highly heterogeneous malignant tumor. Among the various epigenetic alterations involved in its pathogenesis, the *N*^6^-methyladenosine (m^6^A) modification of eukaryotic mRNA plays a pivotal role in tumor biology and holds significant potential for diagnostic and therapeutic applications. In this study, we identified and validated an 8-gene risk score that robustly predicts prognosis in HCC patients, achieving area under the ROC curve (AUC) values greater than 0.75 across both internal and external cohorts. A high-risk score was significantly associated with poorer clinical outcomes and reduced immune tolerance, while also indicating a potentially enhanced response to immunotherapy. Single-cell RNA sequencing revealed a higher proportion of T cells and a decreased presence of immunosuppressive T cells in the high-risk group, which may account for their improved responsiveness to immune checkpoint inhibitors. Finally, in vitro experiments and immunohistochemical staining showed the biological function of the key gene *ANLN*.

## 1. Introduction

There is a high prevalence of hepatocellular carcinoma (HCC), which usually develops upon liver inflammation and chronic liver disease [1]. With an estimated 865,000 new cases globally in 2022, primary liver cancer is responsible for approximately 758,000 deaths annually, ranked as the sixth most common malignancy and the third leading cause of cancer mortality [2,3]. Furthermore, the incidence of HCC continues to rise because of the advancements in diagnostic technologies over the past decade [4]. Despite comprehensive multidisciplinary treatment following hepatectomy, HCC cells are capable of evading these treatments, resulting in cancer recurrence and metastasis [5,6]. The prognosis for HCC patients generally remains poor, with an overall five-year survival rate of less than 20% worldwide [7]. Therefore, the discovery of a stable and reliable tumor classification and molecular features gives ever more weight to assessing patient prognosis and suggesting more effective treatments.

*N*6-methyladenosine (m^6^A) modification is the most prominent and abundant internal modification in messenger RNAs and long non-coding RNAs in nearly all higher eukaryotes [8,9,10]. An m^6^A modification is an epigenetic change with a dynamically reversible process, performed by the methyltransferase complex (“writers”), demethylase (“erasers”), and functional manager (“readers”), which plays an essential role in RNA metabolism, affecting RNA stability, splicing, translation, and translocation [11,12,13,14]. The methyltransferase complex, consisting of methyltransferase-like 3 (METTL3), methyltransferase-like 14 (METTL14), Wilms tumor 1-associating protein (WTAP), vir-like m^6^A methyltransferase-associated protein (KIAA1429, also known as VIRMA), zinc finger CCCH-type containing 13 (ZC3H13), and RNA-binding motif protein 15 (RBM15), catalyzes the methylation of adenine at the nitrogen atom on the sixth carbon of the aromatic ring. Demethylases, such as the fat mass and obesity-associated protein (FTO) and alkB homolog 5 (ALKBH5), selectively remove the methyl group, reversing the modification. Additionally, functional managers—including YT521-B homology domain-containing family proteins 1 and 2 (YTHDF1, YTHDF2), YT521-B homology domain-containing proteins 1 and 2 (YTHDC1, YTHDC2), and heterogeneous nuclear ribonucleoprotein C (HNRNPC)—play a critical role in “reading” RNA methylation signals and regulating downstream RNA degradation.

The epigenetic modifications of m^6^A affect the expression of both oncogenes and suppressor genes, regulating carcinogenesis and tumor progression [15,16]. Recent evidence has shown significantly upregulated METTL3 in gastric cancer, and its high expression is tightly associated with a poor clinical prognosis [17]. In addition, accumulating studies suggest that m^6^A is highly associated with tumor immunophenotypes and antitumor immune responses. Su et al. demonstrated FTO’s essential role in mediating tumor immune evasion [18], and they suggested that it may serve as a promising target for immunotherapy. Moreover, Du et al. developed a prognostic signature based on m^6^A regulators in HCC and identified correlations between certain m^6^A regulators and patient responses to targeted therapies [19], highlighting the pivotal role of m^6^A in shaping the tumor microenvironment. Similarly, Jiang et al. constructed an m^6^A-related gene signature as a biomarker for predicting HCC prognosis and treatment response to sorafenib and anti-PD-1 immunotherapy. Their findings revealed that patients responsive to sorafenib had significantly lower risk scores, while no significant difference was observed in response to anti-PD-1 therapy. However, the predictive accuracy of the model was limited by the relatively small number of m^6^A regulators included, with most area under the ROC curve (AUC) values falling below 0.7. Furthermore, the model showed limited utility in predicting immune checkpoint expression and immunotherapy response. These limitations highlight the need to construct a more robust prognostic model based on a broader set of m^6^A regulator-related genes, aiming to improve the prediction of prognosis and immunotherapeutic efficacy in HCC patients.

We conducted a meticulous screening of m^6^A regulator-related genes in HCC using data from public databases, aiming to explore potential mechanisms. Subsequently, we employed a univariate and least absolute shrinkage and selection operator (LASSO) Cox regression analysis to identify differentially expressed prognostic m^6^A-related genes. With the application of the multivariate Cox regression analysis, we successfully established a risk signature composed of eight m^6^A-related genes. Significantly, we verified the accuracy of this signature in both the training and ICGC testing groups. Furthermore, we investigated the relationships between the prognostic signature, somatic mutation, and immunotherapy responsiveness. The single-cell RNA data of HCC patients allowed for a comprehensive depiction of the cellular composition within the high and low-risk groups. Of particular importance was the in-depth analysis of T cells, revealing a higher proportion of T cells in the high-risk group, with a reduced transition towards heterogeneous T cell states. Additionally, we explored differences in the tumor mutation burden (TMB) and mRNA stemness index (mRNAsi) across different m^6^A clusters. Our findings revealed a significant positive correlation between risk scores, TMB, and mRNAsi, suggesting that variations in m^6^A-related gene expression, potentially driven by mutations such as TP53, contribute to differential prognoses among patient subgroups. Importantly, we conducted in vitro evaluations of the selected gene *ANLN*. In conclusion, our study offers novel insights into the development of stable prognostic tools that have the potential to benefit a substantial number of HCC patients.

## 2. Materials and Methods

### 2.1. Acquisition of Data

The mRNA expression data and relevant clinical information of three independent HCC cohorts (TCGA-LIHC, n = 370; GSE76427, n = 115; and ICGC-LIRI-JP, n = 231) were obtained from three public databases: The Cancer Genome Atlas (TCGA), the Gene Expression Omnibus (GEO), and the International Cancer Genomics Consortium (ICGC). Data collection was fully in line with the usage guidelines of the TCGA, ICGC, and GEO databases. In this study, the training cohort (n = 485) was constituted of TCGA and GSE76427 because of their exhaustive information, and the ICGC dataset formed the testing cohort. For data normalization, the R package “limma” was used to transform the values of the fragments per kilobase of transcript per million (FPKM) data into transcripts per million kilobase (TPM) values in the three RNA-seq cohorts. Meanwhile, using the ComBat function from the R package “SVA” (version 3.56), the batch effect in different datasets was removed.

### 2.2. Human Tissue Samples and Tissue Microarrays

Human hepatocellular carcinoma (HCC) resection specimens were obtained from patients who underwent hepatectomy at Ruijin Hospital (Shanghai, China) between January 2022 and December 2023. The sample collection and preparation protocol were approved by the Ruijin Hospital Ethics Committee (reference number: 2021-421). Written informed consent was obtained from all patients. Additionally, consent to publish relevant clinical information that could potentially identify individuals (age, gender, histological grade, etc.) was obtained. This research was conducted in accordance with the principles of the Declaration of Helsinki. All patients were enrolled if the following conditions were met: (1) the histopathological diagnosis with HCC had been made; (2) there had been no prior anti-cancer treatments; (3) there was no other history of malignancy. The samples were confirmed based on at least two pathologists’ assessments. A total of 57 pairs of normal liver tissue and HCC tissue were obtained from surgical specimens for tissue microarrays, including 46 males and 11 females with median ages of 59 and 58 years old.

### 2.3. Consensus Clustering of m^6^A Regulators

According to the expression levels of 23 recognized m^6^A regulators, including 10 writers (ZC3H13, METTL3, METTL5, METTL14, METTL16, VIRMA, WTAP, RBM15, RBM15B, and CBLL1), 3 erasers (ALKBH3, ALKBH5, and FTO), and 10 readers (LRPPRC, HNRNPA2B1, ELAVL1, YTHDC1, YTHDF1, YTHDF2, YTHDF3, HNRNPC, IGFBP3, and RBMX), unsupervised clustering analysis was performed to identify various m^6^A modification subtypes and patient classification for further analyses. The R package “ConsensusClusterPlus” with a computation cycle of 10,000 times was employed to evaluate cluster numbers and their stabilities [20]. The overall survival (OS) differences between different subtypes were analyzed using the Kaplan–Meier method.

### 2.4. Identification of Differentially Expressed Genes

Using the R package “limma” (FDR < 0.001) to analyze gene expression in different subtypes, DEGs were identified. A principal component analysis (PCA) was visualized by the “ggplot2” R package (version 3.52) to observe the clustering conditions of the m^6^A modification patterns. The m^6^A regulator-related gene expression heatmaps and the DEG expression heatmaps were visualized by the “pheatmap” R package (version 1.0.12).

### 2.5. Construction of a Risk Score for Predicting the Overall Survival of Hepatocellular Carcinoma

DEGs in the training group (n = 485) were screened by a univariate Cox analysis of OS (*p* < 0.001). In order to reduce the scope of the prognosis-related genes, the least absolute shrinkage and selection operator (LASSO) algorithm was used to remove overfitting between the prognosis-related genes, with 10-fold cross-validation based on the R package “glmnet” employed to tune the penalty parameter. Then, the obtained genes with non-zero regression coefficients were further analyzed using multivariate Cox regression. The risk score was established by multiplying the expression level of each gene’s coefficients. The optimal cut-off value was achieved with the “survminer” R package (version 0.5.0), dividing all patients into a high-risk or low-risk group. We then compared the survival curve in each group by the Kaplan–Meier method and the log-rank test, with a *p*-value < 0.05 considered statistically significant [21]. The sensitivity and specificity of our risk score model as a survival predictor were evaluated by receiver operating characteristic (ROC) curves and areas under the ROC curves (AUC values) using the “survival ROC” R package (version 1.0.3.1).

### 2.6. Exploration of the Genomic Features and Stemness Characteristics of the Prognostic Signature

From the TCGA GDC Data Portal, the somatic mutation data were acquired to study the gene mutation rate and TMB across risk groups. mRNAsi is an index which uses gene expression data to quantitatively reflect the stem cell characteristics of cancer cells, with stem cell characteristics growing stronger when their value is close to 1, indicating less differentiated cancer cells [22]. The Kaplan–Meier survival analysis was performed to determine the prognostic impact of gene mutations, TMB, and mRNAsi. Differences in mRNAsi and TMB between the high-risk and low-risk groups were evaluated using an independent samples *t*-test, while their correlation with the risk score was analyzed using the Spearman correlation test.

In all statistical analyses, *p* < 0.05 was considered indicative of statistical significance.

### 2.7. Validation of the Prognostic Signature and Clinical Correlation Analysis

We investigated the association between our risk model and the clinical features of tumors as well as patient survival outcomes to assess the clinical relevance of our findings. Age, T stage, N stage, and TNM stage were included as clinical variables. Differences in overall survival (OS) between high- and low-risk groups were evaluated using Kaplan–Meier survival analysis, performed with the ‘survival’ and ‘survminer’ R packages. The log-rank test was applied to determine statistical significance. Tumor immune dysfunction and exclusion (TIDE) and the immunophenotype score (IPS) were assessed in high-risk and low-risk groups [23].

### 2.8. Single-Cell RNA Sequencing Analysis

In this study, we obtained single-cell RNA sequencing data from the GEO database, specifically the GSE202642 dataset. Initially, we applied the “Seurat” R package (version 5.3.0) to filter and normalize the scRNA-seq data, retaining genes with significant variability for subsequent analyses. Subsequently, we conducted a principal component analysis (PCA) to reduce the dimensionality of these genes, followed by employing UMAP to segregate cells into distinct clusters. Cell type annotation for each cluster was carried out using the “SingleR” (version 2.11.2) and “celldex” (version 1.19.0) R packages, with reference to the Human Primary Cell Atlas Data and additional marker-based information from the CellMarker database [24]. Furthermore, to analyze the pseudotime of T-cell subtypes, we utilized the monocle2 package. This analysis allowed us to explore the differentiation trajectories and identify the associated genes among the different states of T-cell subtypes.

### 2.9. Cell Culture and Transfection

Human HCC cell lines (HepG2, Hep3B, Huh7, HCCLM3, MHCC97H, and SK-Hep-1) and the human normal hepatic cell line LO2 were obtained from the Cell Bank of the Chinese Academy of Sciences (Shanghai, China). Cells were cultured in either Dulbecco’s Modified Eagle’s Medium (DMEM; Meilunbio, Dalian, China) or RPMI-1640 (Meilunbio, Dalian, China), supplemented with 10% fetal bovine serum (FBS; Gibco, Grand Island, NY, USA), 100 U/mL of penicillin, and 100 μg/mL of streptomycin. All cell lines were maintained in a humidified incubator at 37 °C with 5% CO_2_. Mycoplasma contamination in the cell lines was screened by PCR, and the results are presented in Figure S9. The primers and methods used for detection have been described in detail in a previous publication [25]. For in vitro experiments, LV2N-U6-Puro vectors encoding short hairpin RNA (shRNA) targeting ANLN for gene knockdown were transfected into cultured HepG2 cells during the exponential growth phase (Shanghai GenePharma Co., Ltd., Shanghai, China). HepG2 cell transduced with lentivirus were treated with 2 μg/mL of puromycin for 48 h to establish stable cell lines. The shRNA sequences are listed in Table S1.

### 2.10. Quantitative Reverse Transcriptase PCR, Western Blot Analysis, and Immunohistochemistry Assay

Total RNA was extracted from cultured cell lines using TRIzol™ Reagent (Invitrogen, Carlsbad, CA, USA), according to the manufacturer’s protocol. The purity and concentration of RNA were assessed by spectrophotometry (NanoDrop, Thermo Fisher Scientific, Waltham, MA, USA), and 1 μg of total RNA was reverse transcribed into cDNA using a reverse transcription kit (Vazyme, Nanjing, China). Quantitative real-time PCR (qRT-PCR) was performed using AceQ Universal SYBR^®^ qPCR Master Mix (Vazyme, Nanjing, China) on a Bio-Rad CFX96 Real-Time PCR Detection System. GAPDH was used as the internal control for normalization. The primer sequences used for qRT-PCR are listed in Table S1. Relative gene expression was calculated using the 2^−ΔΔCt^ method.

For Western blotting, total protein was extracted from the HCC cell lines using RIPA lysis buffer (Beyotime, Shanghai, China), which contains 50 mM Tris-HCl (pH 7.4), 150 mM NaCl, 1% NP-40, 0.5% sodium deoxycholate, and 0.1% SDS, supplemented with Complete Mini (Roche, Basel, Switzerland), PhosSTOP (Roche, Basel, Switzerland), 1 mM orthovanadate, and 1 mM phenylmethylsulfonyl fluoride (Pefablock, Roche, Basel, Switzerland). Lysates were incubated on ice for 30 min, followed by centrifugation at 12,000× *g* for 15 min at 4 °C to collect the supernatants. Isolated protein was quantified using a Bradford assay (Bio-Rad, Hercules, CA, USA).

Equal amounts of protein (10–40 μg) were separated on 10% SDS-polyacrylamide gels and transferred to PVDF membranes (Millipore, Burlington, MA, USA) using a semi-dry transfer system. Membranes were blocked with 5% non-fat milk in TBST (20 mM Tris-HCl, 150 mM NaCl, and 0.1% Tween-20, pH 7.6) for 1 h at room temperature and then incubated overnight at 4 °C with the following primary antibodies: anti-Anillin (Abcam, Cambridge, UK, ab252881, 1:1000), anti-GAPDH (Abcam, ab9485, 1:5000), and anti-β-actin (Abcam, ab8226, 1:1000). After washing, membranes were incubated for 1 h at room temperature with HRP-conjugated secondary antibodies: anti-rabbit (Cell Signaling Technology, Danvers, MA, USA, 1:5000) or anti-mouse (Santa Cruz, Santa Cruz, CA, USA, 1:5000). Protein bands were visualized using enhanced chemiluminescence (ECL) reagents (Bio-Rad, USA) and quantified with ImageJ software (version 1.54p).

IHC was performed as previously described [26]. The percentage scores were defined by the percentage of positive cells: 0 (0%), 1 (1–30%), 2(31–60%), 3(61–100%). The staining intensity was classified as intensity scores: 0 (no staining), 1 (light brown staining), 2 (brown staining), 3 (dark brown staining). The total score equals the percentage score multiplied by the intensity score and falls into two categories: weak positive (0–3), strong positive (≥4).

### 2.11. Cell Proliferation Assays

The HCC cells’ proliferation capacity was detected by a colony formation assay (1000 cells/well in six-well plates). In addition, A 5-Ethynyl-20-Deoxyuridine (EdU, Beyotime Biotechnology, Shanghai, China) assay was performed according to the manufacturer’s instructions by the EdU Cell Proliferation Kit with Alexa Fluor 594 (Beyotime Biotechnology, Shanghai, China).

### 2.12. Cell Migration Assays

Stably transfected HCC cells (5 × 10^4^ cells/100 µL) suspended in serum-free medium were plated in the top chambers. The lower chambers contained 700 µL of DMEM supplemented with 10 % FBS. After 24 h, the migrated HCC cells were fixed at room temperature for 20 min with a 1% crystal violet stain solution and were counted manually. For wound healing assays, 3 × 10^5^ cells were seeded into each well of a 6-well plate and cultured for 24 h to allow them to reach confluence. A wound was then created by scraping the cell monolayer with a 200-μL pipette tip. After wounding, the cells were washed to remove debris and cultured in serum-free medium for an additional 24 and 48 h. Wound closure was monitored and photographed at the indicated time points.

### 2.13. Statistical Analysis

All statistical analyses were performed using R software (version 4.1.3) and GraphPad Prism 10 (GraphPad Software, La Jolla, CA, USA). For comparisons between two groups, an unpaired two-tailed Student’s *t*-test was used. For comparisons among more than two groups, one-way ANOVA was applied, followed by Tukey’s post hoc test to assess pairwise differences. A *p*-value < 0.05 was considered statistically significant.

## 3. Results

### 3.1. Consensus Clustering of m^6^A Genes in Two Clusters with Different Clinical Outcomes of HCC

**Figure 1:**
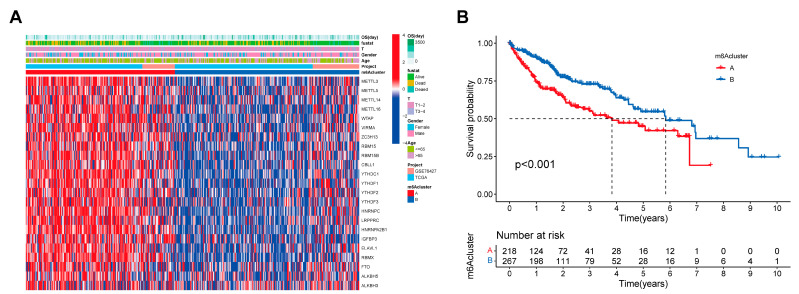
Clustering analysis based on the expression of 23 m^6^A regulator-related genes in a combined cohort of 485 HCC patients identified two distinct m^6^A clusters with significant differences in overall survival. (**A**) Heatmap of m^6^A regulator gene expression showing two clusters: a red cluster with high m^6^A-related gene expression and a blue cluster with low expression. (**B**) Kaplan–Meier overall survival curves showing a significantly poorer prognosis in the high m^6^A-related gene expression group (n = 218) compared to the low expression group (n = 267), based on the median expression cutoff (*p* < 0.001).

First, we performed consensus clustering analysis on the expression levels of 23 m^6^A regulator genes in HCC patients to explore their association with HCC subtypes. The optimal clustering was achieved when the consensus matrix k-value was set to 2 (Figure S1A), resulting in the lowest intergroup correlation and highest intragroup correlation. This finding suggests that the 23 m^6^A regulators effectively classified the training cohort (n = 485, merged from TCGA-LIHC and GSE76427) into two distinct subtypes (Figure 1A).

The Kaplan–Meier survival analysis indicated that patients in Cluster B had a significantly better OS than those in Cluster A (Figure 1B). A principal component analysis (PCA) further confirmed that the expression levels of the 23 m^6^A regulators were clearly distinguishable between the two clusters (Figure S1B). Additionally, a differential expression analysis identified significant DEGs between the subtypes (Figure S1D), with 10,780 intersecting DEGs (Figure S1C).

### 3.2. An 8-Gene Prognostic Signature Established in the Training Cohort

**Figure 2:**
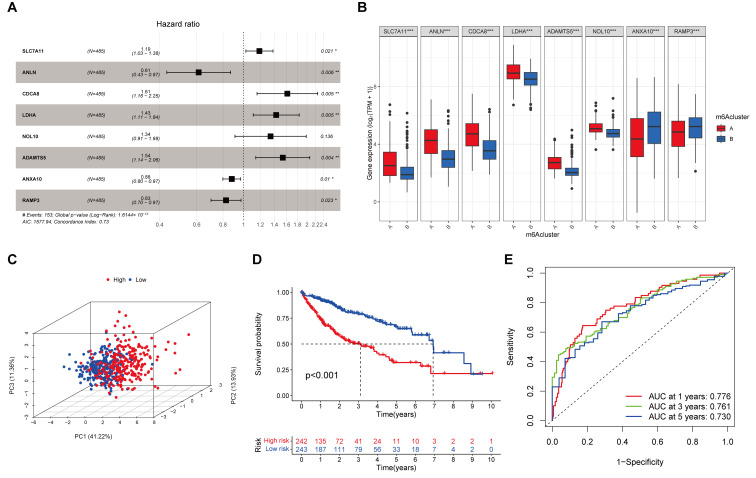
Evaluation of the m^6^A-related gene prognostic model in the training cohort (TCGA-LIHC merged with GSE76427). (**A**) A prognostic model comprising eight m^6^A-related genes was constructed using multivariate Cox regression analysis (*p* < 0.05 *, *p* < 0.01 **). (**B**) Boxplot comparing the expression levels of the eight prognostic genes between the low m^6^A regulator gene expression cluster and the high m^6^A regulator gene expression cluster (*p* < 0.001 ***). (**C**) 3D PCA plot showing the separation between high-risk and low-risk groups. (**D**) Kaplan–Meier overall survival curves demonstrating a significantly poorer prognosis in the high-risk group (n = 242) compared to the low-risk group (n = 243), based on the median risk score cutoff (*p* < 0.001). (**E**) Time-dependent ROC analysis assessing the predictive performance of the risk score for overall survival in the training cohort, with AUC values greater than 0.75.

A total of 1312 intersecting prognosis-associated DEGs were selected by the univariate Cox regression analysis (*p* < 0.001, Table S2). From this, we sorted out genes with non-zero LASSO regression coefficients (Figure S2A,B). Ultimately, a risk score was built with eight genes by the multivariate Cox regression analysis: SLC7A11 × 0.1742 − ANLN × 0.4893 + CDCA8 × 0.4790 + LDHA × 0.3571 + ADAMTS5 × 0.4293 + NOL10 × 0.2960 − ANXA10 × 0.1277 − RAMP3 × 0.1905 (Figure 2A). The expression levels of these eight genes differed significantly between the two subtypes (Figure 2B). Based on their hazard ratio (HR) values, we categorized them into risk genes (SLC7A11, CDCA8, LDHA, ADAMTS5, and NOL10) and protective genes (ANLN, ANXA10, and RAMP3).

Additionally, a correlation analysis revealed a strong association between the 23 m^6^A regulators and the risk scores derived from these eight genes (Figure S2C). Furthermore, the Kaplan–Meier survival analysis confirmed that these eight risk genes serve as essential prognostic factors, as shown in Figure S3.

### 3.3. Evaluation and Validation of the Prognostic Signature

As described in the risk model above, the training and test groups were divided into low-risk and high-risk groups based on the median value of risk scores in the training cohort. The PCA analysis (Figure 2C and Figure S4A) demonstrated that individuals with different risk levels were distinguishable. The survival curves (Figure 2D and Figure S4B) showed a favorable survival rate in the low-risk group (*p* < 0.001). In the training cohort, the area under the curve (AUC) values for 1-year, 3-year, and 5-year OS predicted by the risk scores were 0.776, 0.761, and 0.730, respectively, indicating a solid separation capability (Figure 2E). Similarly, in the ICGC testing group (Figure S4C), the AUC values of the 8-gene signature were 0.758, 0.783, and 0.796 for 1, 3, and 5 years, respectively.

To evaluate the applicability of the 8-gene signature as a survival prediction tool across different clinical characteristics, a stratification analysis was conducted based on age (≤65 and >65), grade, T stage, M stage, N stage, and TNM stage (Table 1). The results demonstrated a significant improvement in OS for the low-risk group in all subgroups (n = 10) (Figure S5), indicating that the 8-gene signature is an effective prognostic tool for patients with HCC.

### 3.4. The Benefit of ICIs Therapy in Different Risk Subgroups

**Figure 3:**
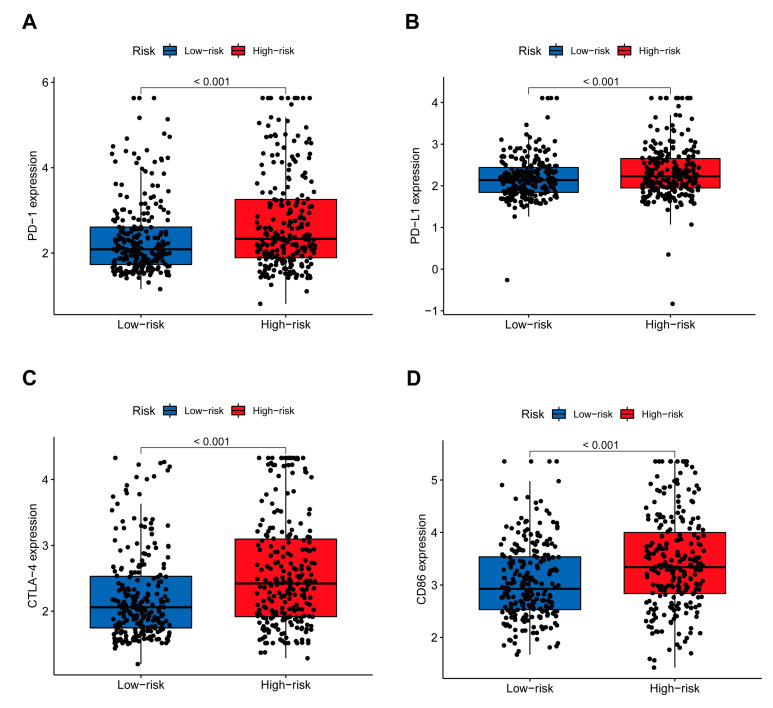
Relationship between risk scores and immune checkpoint expression. (**A**) Expression levels of PD−1 in high-risk (n = 242) and low-risk (n = 243) groups, showing significantly higher expression in the high-risk group (fold change = 0.40, *p* < 0.001). (**B**) Expression levels of PD−L1, with significantly higher expression in the high-risk group (fold change = 0.17, *p* < 0.001). (**C**) Expression levels of CTLA−4, with significantly higher expression in the high-risk group (fold change = 0.36, *p* < 0.001). (**D**) Expression levels of CD86, with significantly higher expression in the high-risk group (fold change = 0.39, *p* < 0.001).

Immunotherapy may offer a promising treatment option for unresectable HCC patients by halting tumor progression. To predict immunotherapy responses utilizing the 8-gene signature, we conducted a correlation analysis of risk scores with common immune checkpoints (ICPs), such as programmed cell death 1 (PDCD1), CD274 (PD-L1), cytotoxic T lymphocyte-associated protein 4 (CTLA4), CD86 (Figure 3A–D), CD28, T cell immunoglobulin and mucin-containing molecule 3 (TIM-3), galectin-9 (GAL-9), lymphocyte-activating 3 (LAG3), T cell immunoreceptor with Ig and ITIM domains (TIGIT), CD200, and CD200R1 (Figure S6A–G). Among these, PDCD1 (fold change = 0.40, *p* < 0.001), CD274 (fold change = 0.17, *p* < 0.001), CTLA4 (fold change = 0.36, *p* < 0.001), and CD86 (fold change = 0.39, *p* < 0.001) showed significantly higher expression levels in the high-risk group compared to the low-risk group. Similar trends were observed for other ICPs, suggesting enhanced immune activation and potential susceptibility to ICPs in high-risk patients. These findings highlight the possible role of the 8-gene signature in predicting responsiveness to immunotherapy.

Moreover, to evaluate the immune response of HCC patients, we calculated TIDE scores and IPS to predict the patients’ response capability (Figure S6H–L). Higher TIDE prediction scores indicate immune evasion and a lower likelihood of benefiting from immune checkpoint inhibitor (ICI) therapy. The results revealed that the TIDE score was lower in the high-risk group than in the low-risk group, suggesting that high-risk HCC patients may have a more favorable ICI therapy outcome than low-risk patients (Figure S6H). To verify this idea, we compared the effects of anti-CTLA4 and anti-PD1 treatments between different risk subgroups (Figure S6I–L). Both anti-CTLA4 treatment and anti-PD1 immunotherapy were found to perform better in patients with high-risk scores.

### 3.5. Exploration of m^6^A-Related Risk Genes in the Single-Cell Level

We collected single-cell RNA sequencing (scRNA-seq) data from 7 HCC samples within the GSE202642 dataset. After implementing data filtering and normalization procedures, we carefully selected the top 3000 genes with the highest variance for subsequent cell classification. The expression levels of these 3000 genes were then subjected to PCA dimensionality reduction, resulting in PC1-20. Subsequently, we performed UMAP dimensionality reduction and visualization on PC1-20, leading to the segregation of all cells into 33 distinct clusters. Cell annotation was meticulously conducted for each cluster, revealing major cell types, including T cells, B cells, Natural Killer (NK) cells, epithelial cells, hepatocytes, macrophages, and monocytes (Figure S7A).

Furthermore, to delve deeper into the differences between the low-risk and high-risk groups, we meticulously analyzed the proportions of immune cell subtypes in the low risk and high-risk groups, as illustrated in Figure S7B. In comparison to the low-risk group, the high-risk group exhibited higher proportions of T cells, endothelial cells, and dendritic cells, while displaying fewer macrophages. Additionally, we conducted a more detailed analysis of T cell subtypes, primarily consisting of CD4^+^ Tem, CD8^+^ Tem, Tregs, and CD4^+^ T cells (Figure S7C). Through comprehensive cell trajectory analysis, we identified the risk model genes as determinants of T cell differentiation states. Importantly, the elevated expression of low-risk genes was found to promote differentiation towards heterogeneous T cell states, including Tregs (Figure S7D,E).

### 3.6. Association of Risk Score with Gene Mutation, Tumor Mutation Burden, and mRNA Stemness Index

The frequent occurrence of tumor suppressor gene mutations, particularly TP53 alterations in cancer patients, prompted our investigation into the distinct prognostic outcomes and immune microenvironment characteristics across different m^6^A clusters. Our analysis focused on the interrelationships between TP53 mutation status, TMB, mRNAsi, and risk scores. To elucidate the difference in gene mutation between the high-risk and low-risk groups, oncoplots were utilized. Notably, a higher incidence of TP53 mutations was observed in the high-risk group compared to the low-risk group (43% vs. 13%, Figure S8A,B). Previous studies have identified the TMB and mRNA stemness index (mRNAsi) as key indicators of tumor immune response, with higher TMB and mRNAsi suggesting an increased likelihood of benefiting from immune checkpoint inhibitor (ICI) therapy [22]. In accordance with these findings, our analysis demonstrated that patients with TP53 mutations, higher TMB, and higher mRNAsi exhibited significantly worse prognoses (Figure S8C,F,I).

Furthermore, the high-risk group showed elevated TMB and mRNAsi levels (Figure S8D,G), suggesting that these patients might derive greater benefit from immunotherapy. The Spearman correlation analysis further confirmed positive correlations between risk scores and TMB/mRNAsi (Figure S8E,H).

### 3.7. ANLN Inhibits HCC Cell Proliferation and Migration In Vitro

**Figure 4:**
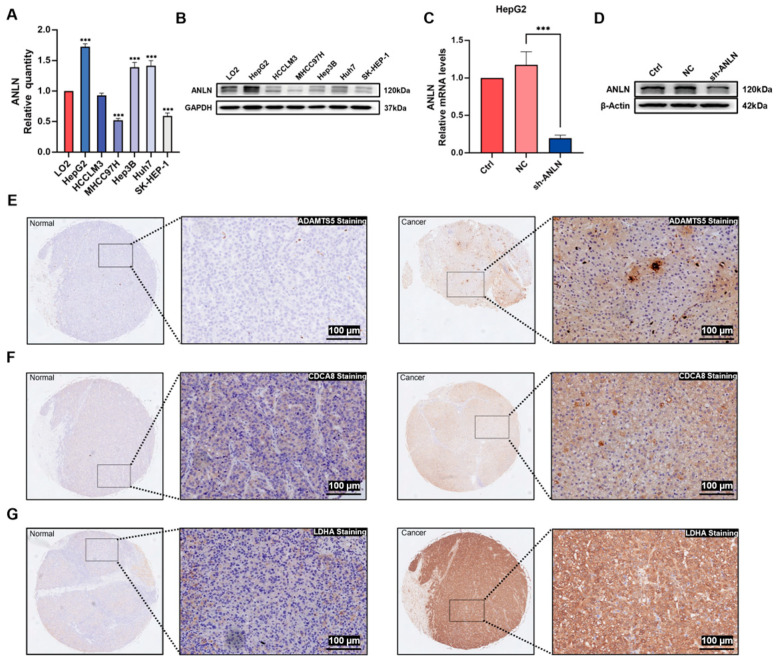
Expression of model genes in HCC cell lines and clinical tissues and establishment of an ANLN-Knockdown HCC cell line. (**A**) Comparative qRT-PCR analysis of ANLN mRNA expression levels in six HCC cell lines versus the normal hepatocyte line LO2. (**B**) Western blot analysis of ANLN protein expression profiles in HCC cell lines and LO2 control cells. (**C**) Quantitative assessment of ANLN mRNA knockdown efficiency by qRT-PCR in HepG2 cells following transfection with sh-ANLN. (**D**) Western blot validation of ANLN protein downregulation in sh-ANLN-transfected HepG2 cells compared with control. (**E**–**G**) Representative immunohistochemical staining patterns of AMADTS5, CDCA8, and LDHA in HCC specimens and matched adjacent normal liver tissues. Data represent mean of n = 3 ± SD; *** *p* < 0.001 (One-way ANOVA). The uncropped blots are shown in Figure S10.

**Figure 5:**
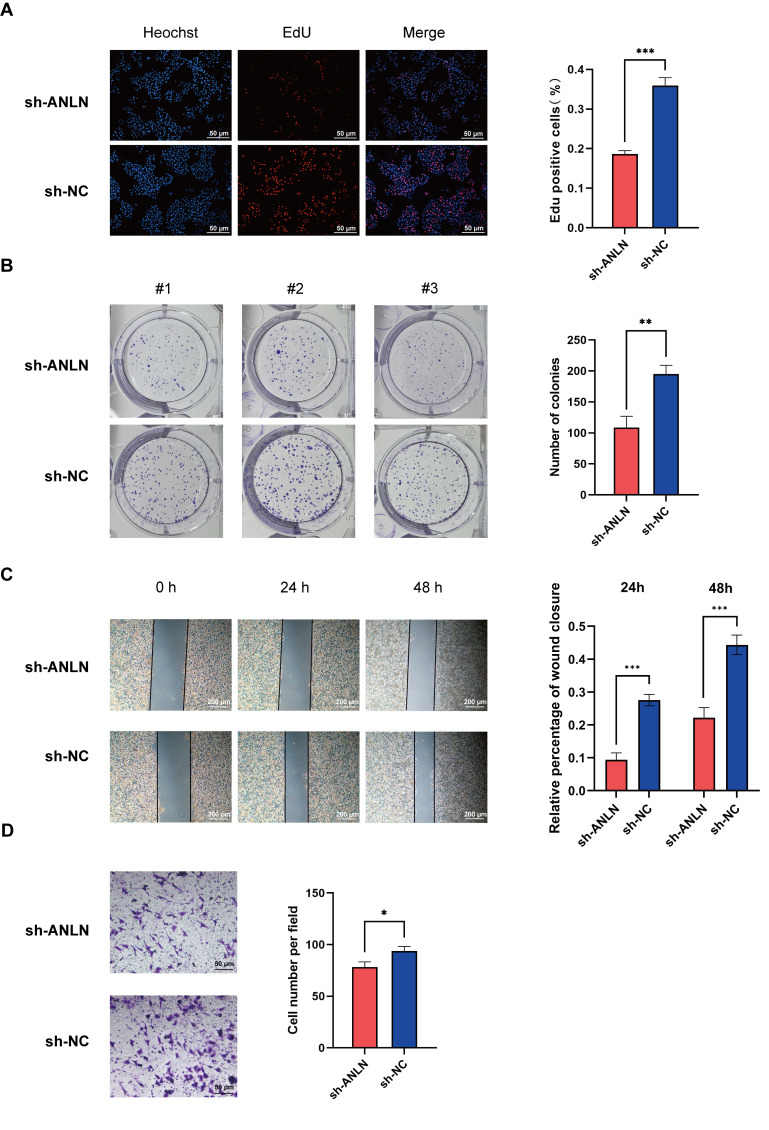
ANLN promotes the proliferation and migration of HCC cells. (**A**) EdU incorporation assay assessing the proliferative capacity of HepG2 cells following ANLN knockdown, with significantly fewer EdU-positive cells observed in the sh-ANLN group compared to the NC group. (**B**) Colony formation assay showing decreased clonogenic ability in HepG2 cells transfected with sh-ANLN, as evidenced by fewer and smaller colonies. (**C**) Wound healing assay indicating impaired migratory potential in sh-ANLN-transfected HepG2 cells, with reduced wound closure compared to the control group at both 24 and 48 h. (**D**) Transwell migration assay quantifying the invasive capacity of HepG2 cells after ANLN knockdown, with significantly fewer migrated cells in the sh-ANLN group. Data represent mean of n = 3 ± SD; * *p* < 0.05, ** *p* < 0.01, *** *p* < 0.001 (unpaired two-tailed Student’s *t*-test).

To elucidate the role of *ANLN*, an essential gene in our risk model, in HCC, we first selected HepG2 cells with the highest level of *ANLN* mRNA among six HCC cells and downregulated its expression using RNA interference (Figure 4A–D). We then evaluated the effect of *ANLN* on HCC cell proliferation through EdU, colony formation, and CCK-8 assays, which revealed that *ANLN* knockdown inhibited HCC cell proliferation (Figure 5A,B). We further confirmed that the downregulation of *ANLN* expression inhibited the migration and invasion ability of HepG2 cells using wound healing tests and Transwell assay (Figure 5C,D). Additionally, we analyzed the expression of ADAMTS5, CDCA8, and LDHA, the top three high-risk genes, in a liver cancer tissue array comprising 57 paired liver cancer tissues. The results showed an increased expression of ADAMTS5, CDCA8, and LDHA in tumor tissues, suggesting their significant regulatory roles in HCC (Figure 4E–G).

## 4. Discussion

Primary liver cancer is a prevalent malignancy with high morbidity and mortality rates, making it a global health issue. HCC is the most common type of liver cancer, accounting for 75% of cases. Its occurrence is linked to viral hepatitis, alcoholic fatty liver, and non-alcoholic fatty liver diseases [27]. Since 2017, immunotherapy has emerged as a breakthrough treatment for advanced liver cancer [28]. CheckMate040 and KEYNOTE-224 have established the second-line treatment status of nivolumab and pembrolizumab in liver cancer, respectively, and are currently being investigated for their impact on first-line treatment [29,30]. Results from the nivolumab study in CheckMate459 have shown that its objective response rate and complete response rate are superior to sorafenib [31]. Clinical data have demonstrated that ICI treatment is effective, and some liver cancer patients can achieve lasting disease control [28]. Therefore, it is of great significance to researchers to better utilize biomarkers to predict patient prognosis and assist clinicians in developing immunotherapy strategies.

The dysregulation of m^6^A methylation regulators has been demonstrated to be associated with the occurrence and progression of various tumors. m^6^A RNA methylation, which is regulated by m^6^A methyltransferases, demethylases, and binding proteins, is the most common modification affecting cancer progression [15]. Zhang et al. reported that YTHDF2, a reader of m^6^A, promoted liver cancer metastasis by mediating the m^6^A methylation of OCT4 mRNA in hepatocellular carcinoma [32]. Similarly, Chen et al. [33] found that METTL3, a major RNA N6-adenosine methyltransferase, is significantly upregulated in liver cancer and is associated with a poor prognosis. Moreover, the encouraging results from METTL3 inhibitors, several of which are currently in early clinical development, along with evidence that combining METTL3 inhibitors with PD-L1 blockade enhances antitumor immune efficacy, offer new avenues for cancer treatment [34]. Understanding the role of m^6^A methylation regulators in liver cancer is thus crucial for developing effective therapeutic strategies. However, current prognostic models suffer from limited accuracy due to the small number of m^6^A regulators employed [35]. Consequently, efforts have been made to construct prognostic models using a larger pool of m^6^A regulator-related genes to improve the prediction of prognosis and immunotherapy response in HCC patients.

In this study, we employed a consensus clustering analysis based on the expression levels of 23 m^6^A regulatory factors to stratify HCC patients into two clusters, which exhibited significant differences in overall survival OS. Our findings indicate a strong correlation between m^6^A regulator expression and both the prognosis and malignancy of HCC. Furthermore, we identified DEGs between the group with high expression of m^6^A-related genes and the group with low expression, to explore their molecular differences. Notably, 1312 overlapping m^6^A-related DEGs were found to be prognostically significant.

Subsequent univariate and LASSO Cox regression analyses allowed us to construct a risk model based on eight novel m^6^A-related genes—SLC7A11, CDCA8, LDHA, ADAMTS5, NOL10, ANLN, ANXA10, and RAMP3—derived from their multivariate Cox regression coefficients. HCC patients classified as high-risk according to this 8-gene signature experienced significantly poorer OS in both the training and ICGC testing groups. Moreover, a time-dependent ROC curve analysis demonstrated the high predictive accuracy of our risk model, with AUC values consistently exceeding 0.75. These findings suggest that the 8-gene risk score may serve as a clinically useful tool to inform surveillance intensity, therapeutic decision-making, and long-term patient management in HCC.

SLC7A11 is a component of the cystine/glutamate antiporter xCT and is closely associated with ferroptosis. Recently, it has been discovered that SLC7A11 is also intimately linked with m^6^A modification. Chen et al. found that METTL3 can directly regulate the transcription of the ferroptosis regulator SLC7A11 via the m^6^A/IGF2BP2 pathway, thereby promoting radiation resistance [36]. In addition, Shuai et al. discovered that a RBM15-mediated m^6^A modification of LDHA mRNA enhances the stability of LDHA expression, playing a tumorigenic role in lung adenocarcinoma progression [37]. ANLN, a well-known cytoskeletal protein, plays a critical role in hepatocyte polyploidization. ANLN is also closely associated with m^6^A modification; Hao et al. [38] found that the upregulation of METTL3 enhances m^6^A modification on ANLN mRNA, while YTHDF1 directly binds to the m^6^A sites within ANLN mRNA to maintain its stability, thereby promoting bone metastasis in HCC.

Importantly, our study also examined differences in the tumor immune microenvironment between the risk groups and their implications for immunotherapy response. We observed significant variations in the expression of immune checkpoint-related genes across the groups, highlighting the importance of patient risk stratification when devising immunotherapy strategies. Considering the pivotal role of immune cell infiltration as a predictive factor for the response to ICIs [39], we meticulously examined the association and potential predictive value of the risk score in immunotherapy. We meticulously analyzed the expression of several well-established immune checkpoint inhibitor genes in different risk groups and assessed the response to ICIs using TIDE and IPS features. Our research findings suggest that HCC patients in the high-risk group exhibit a lower TIDE score, which may indicate a positive response to anti-PD1 and anti-CTLA-4 therapy. Moreover, single-cell sequencing data further validated our model’s classification, enhancing its utility in predicting patient responses to immunotherapy. We found that the high-risk group exhibited higher proportions of T cells, endothelial cells, and dendritic cells, while the proportion of macrophages was lower. This disparity may partly explain the increased T cell activity and decreased immunosuppressive macrophage presence in the high-risk group, thereby enhancing sensitivity to immunotherapy. Furthermore, a comprehensive cellular trajectory analysis of T cell subtypes—including CD4^+^ Tem, CD8^+^ Tem, Tregs, and CD4^+^ T cells—further demonstrated that the risk model genes play a decisive role in T cell differentiation. Notably, an elevated expression of low-risk genes appears to promote differentiation toward a heterogeneous T cell state, including Tregs, which may in turn impair the efficacy of immunotherapy, consistent with our model predictions. Collectively, these findings suggest that the risk score could serve as a predictive biomarker to identify HCC patients more likely to benefit from immunotherapy, thereby facilitating personalized treatment strategies.

In this 8-gene risk model, ANLN exhibited the highest positive coefficient (0.4893), indicating that a higher expression of this gene is associated with an increased risk of death or adverse events. We further validated its biological mechanism by generating a stable knockdown HCC cell line, which showed that *ANLN* suppression significantly impaired HCC cell proliferation and migration. Additionally, we assessed whether *ANLN* knockdown altered the expression levels of the other seven genes in the model. Unfortunately, no meaningful changes were observed. This suggests that each gene in our model contributes independently to overall survival, and that the model contains minimal multicollinearity among its components.

However, our study is limited by the retrospective nature of the data obtained from public databases, which may introduce inherent selection bias. Although we performed IHC validation of the model genes using our in-house patient cohort, further verification was constrained by the limited availability of long-term prognostic data. Moreover, we did not conduct mechanistic investigations into the role of each gene in the progression of HCC. Consequently, high-quality prospective studies, along with complementary in vivo and in vitro experiments, are needed to confirm our findings regarding the relationship between risk scores and ICIs.

## 5. Conclusions

In conclusion, our study provides a good prognostic risk model for HCC patients. The reliability and validity of the signature were validated in multiple datasets. Importantly, the risk model based on the m^6^A molecular subtype can be used for stem cell signature assessment, clinical signature evaluation, immune microenvironment evaluation and immunotherapy efficacy prediction in HCC.

## Figures and Tables

**Table 1:** Baseline data of the HCC patients used in this study.

	TCGA-LIHC	GSE76427	LIRI-JP
Age (%)			
≤65	141 (37.4%)	65 (56.5%)	98 (37.7%)
>65	235 (62.3%)	50 (43.5%)	162 (62.3%)
Unknown	1 (0.3%)	NA	NA
Gender (%)			
Female	122 (32.4%)	22 (19.1%)	68 (26.2%)
Male	255 (67.6%)	93 (80.9%)	192 (73.8%)
Stage (%)			
I	175 (46.4%)	55 (47.8%)	40 (15.4%)
II	87 (23.1%)	35 (30.4%)	117 (45.0%)
III	86 (22.8%)	21 (18.3%)	80 (30.8%)
IV	5 (1.3%)	4 (3.5%)	23 (8.8%)
Unknown	24 (6.4%)	NA	NA
Grade (%)			
Grade 1	55 (14.6%)	NA	NA
Grade 2	180 (47.7%)	NA	NA
Grade 3	124 (32.9%)	NA	NA
Grade 4	13 (3.4%)	NA	NA
Unknown	5 (1.3%)	NA	NA

## Data Availability

The data that support the findings of this study are available from the corresponding author upon reasonable request.

## Supplementary Materials

The following supporting information can be downloaded at: https://www.mdpi.com/article/10.3390/cancers17132220/s1, Figure S1: Cluster analysis identifying two distinct m^6^A clusters. Figure S2: Development of the m^6^A-related gene prognostic model in the training cohort (TCGA-LIHC merged with GSE76427). Figure S3: Kaplan–Meier overall survival curves showing statistically significant differences in prognosis based on the expression of the eight prognostic genes. Figure S4: Validation of the m^6^A-related prognostic model in the ICGC testing cohort. Figure S5: Kaplan–Meier survival curves of the eight-gene signature in the training cohort stratified by different clinical characteristics: Figure S6: Associations between risk scores, immune checkpoint expression, and therapeutic sensitivity. Figure S7: Differences in immune cell composition between risk groups and the association of prognostic genes with T cell differentiation. Figure S8: Comprehensive analysis of the risk score in HCC patients. Figure S9: Mycoplasma PCR results for seven cell lines. Figure S10: The original Western blot images. Table S1: The shRNA sequences and primer sequences used in this study. Table S2: Prognostic analysis of 1312 subtype-associated genes with a univariate Cox regression model.

## Abbreviations

The following abbreviations are used in this manuscript:

ANLN	Actin-binding protein
ALKBH5	AlkB homolog 5
AUC	Area under the ROC curve
PD-L1	Programmed death-ligand 1
CTLA4	Cytotoxic T-lymphocyte-associated protein 4
DEGs	Differentially expressed genes
ECL	Enhanced chemiluminescence
EdU	Ethynyl-2′-deoxyuridine
FPKM	Fragments per kilobase of transcript per million mapped reads
FTO	Fat mass and obesity-associated protein
GAL-9	Galectin-9
GEO	Gene Expression Omnibus
HCC	Hepatocellular carcinoma
HNRNPC	Heterogeneous nuclear ribonucleoprotein C
HR	Hazard ratio
ICGC	International Cancer Genomics Consortium
ICIs	Immune checkpoint inhibitors
IPS	Immunophenotype score
LAG-3	lymphocyte-activating 3
LASSO	Least absolute shrinkage and selection operator
m^6^A	*N*6-methyladenosine
METTL	Methyltransferase-like
mRNAsi	mRNA stemness index
NK cells	Natural killer cells
OS	Overall survival
PCA	Principal component analysis
PDCD1	Programmed cell death protein 1
RBM15	RNA-binding motif protein 15
ROC	Receiver operating characteristic
shRNA	Short hairpin RNA
TCGA	The Cancer Genome Atlas
TIDE	Tumor immune dysfunction and exclusion
TIGIT	T cell immunoreceptor with Ig and ITIM domains
TIM-3	T cell immunoglobulin and mucin-domain containing-3
TMB	Tumor mutation burden
TPM	Transcripts per million kilobase
UMAP	Uniform Manifold Approximation and Projection
VIRMA	Vir-Like m^6^A Methyltransferase Associated protein
WTAP	Wilms tumor 1-associating protein
YTH	YT521-B homology
ZC3H13	Zinc finger CCCH-type containing 13
