# Supplementary material for: Stratification of Hepatocellular Carcinoma Using N6-Methyladenosine
Source: Cancers (Basel). 2025 Jul 2;17(13):2220. doi: 10.3390/cancers17132220 (PMC12249396; doi:10.3390/cancers17132220)
Supplement: Supplementary file 1 [file cancers-17-02220-s001.zip › cancers-3699019-supplementary/Supplementary_Figures.pdf]

Supplementary Figures

Stratification of Hepatocellular Carcinoma Using N6-Methyladenosin

Nan Wang[1,2], Jiaxin Shi[3,4], Matthias Bartneck[1], Edgar Dahl[3,4], Junqing Wang[2]

Table of contents

Figure S1. Cluster Analysis Identifying Two Distinct m^{6}A Clusters..**Error! Bookmark not defined.**

Figure S2. Development of the m^{6}A-related gene prognostic model in the training cohort (TCGA-LIHC merged with GSE76427). .... **Error! Bookmark not defined.**

Figure S3. Kaplan–Meier overall survival curves showing statistically significant differences in prognosis based on the expression of the eight prognostic genes.**Error! Bookmark not defined.**

Figure S4. Validation of the m^{6}A-related prognostic model in the ICGC testing cohort .... **Error! Bookmark not defined.**

Figure S5. Kaplan–Meier survival curves of the eight-gene signature in the training cohort stratified by different clinical characteristics .... **Error! Bookmark not defined.**

Figure S6. Associations between risk scores, immune checkpoint expression, and therapeutic sensitivity. .... **Error! Bookmark not defined.**

Figure S7. Differences in immune cell composition between risk groups and the association of prognostic genes with T cell differentiation.. 8

Figure S8. Comprehensive analysis of the risk score in HCC patients.................................. 9

Figure S9. Mycoplasma PCR results for seven cell lines.. 10

Figure S10. The original Western blot images... 11

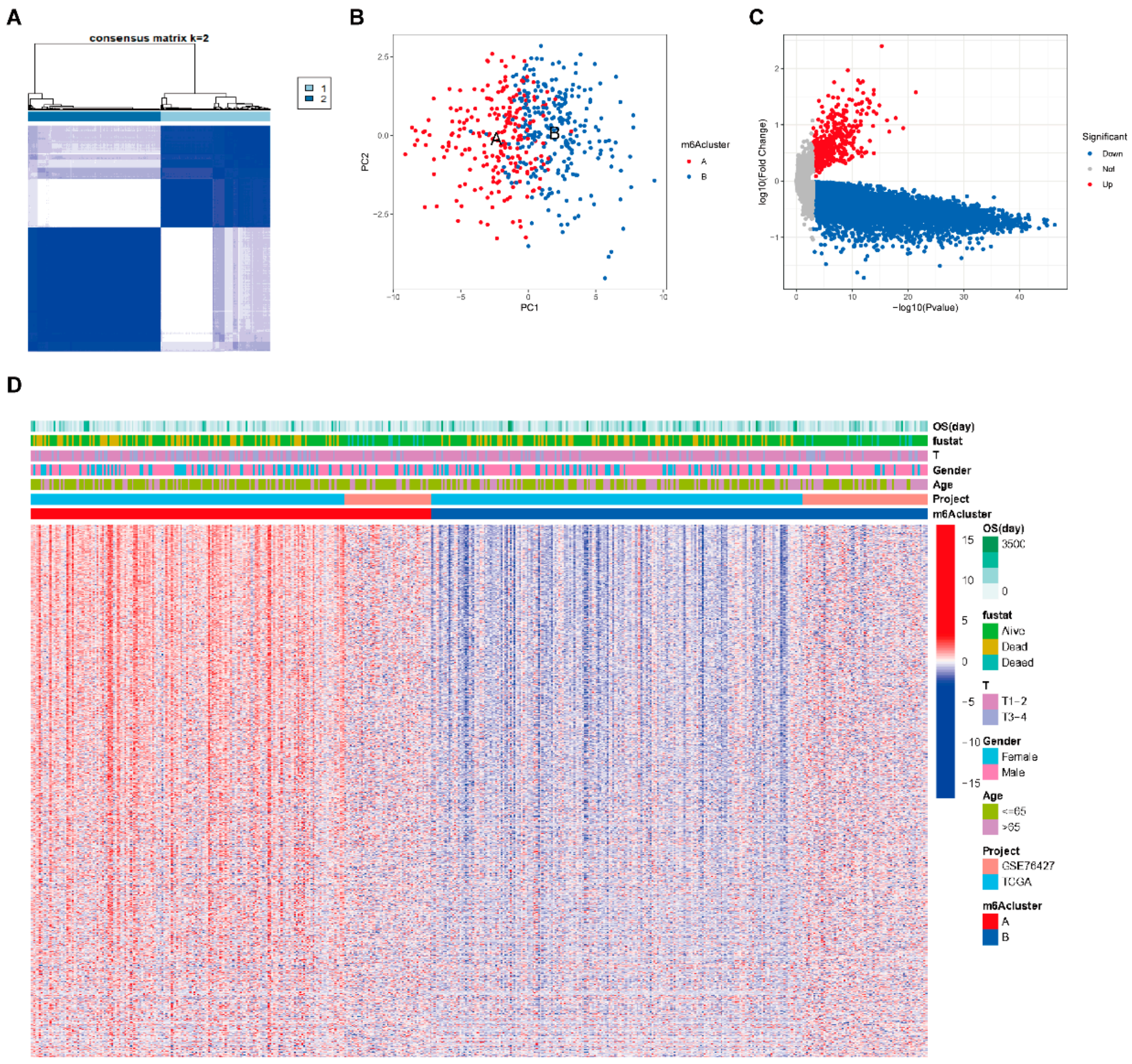

Figure S1. Cluster analysis identifying two distinct m^{6}A clusters

(A) Consensus clustering matrix for k = 2, identifying two stable m^{6}A clusters. (B) Principal component analysis (PCA) showing clear separation between the two m^{6}A clusters: a red cluster with high m^{6}A-related gene expression and a blue cluster with low expression. (C) Volcano plot displaying differentially expressed genes (DEGs)

between the two m^{6}A clusters (Fold change = 1, –Log$_{10}$ *p*-value = 2). (D) Heatmap showing the expression patterns of 10,780 DEGs between the two m^{6}A clusters.

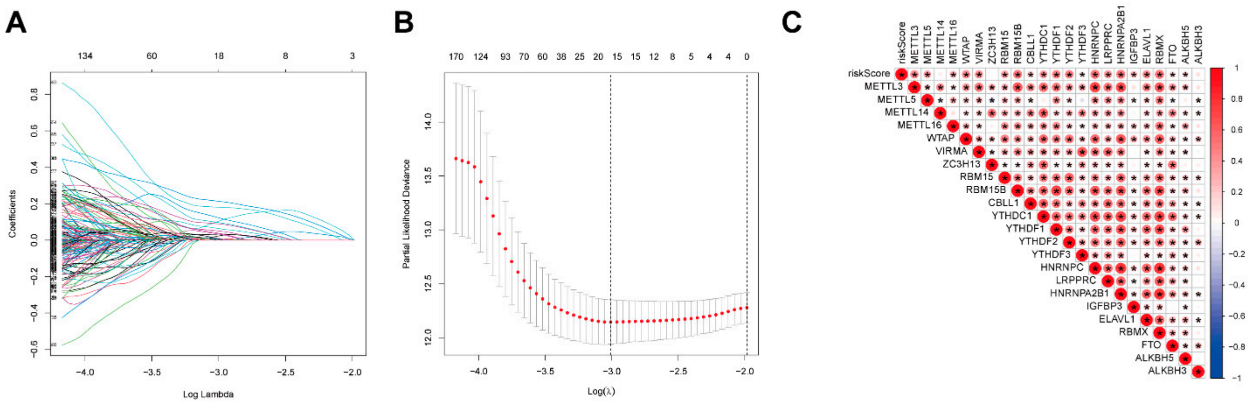

Figure S2. Development of the m^{6}A-related gene prognostic model in the training cohort (TCGA-LIHC merged with GSE76427).

(A) Ten-fold cross-validation for selecting the optimal tuning parameter (λ) in the LASSO regression model. Each curve represents the performance of an individual m^{6}A-related gene. (B) Plot of binomial deviance versus the logarithm of the regularization parameter (log(λ)), with values ranging from −4 to −2. (C) Correlation analysis between risk scores and m6A regulator gene expression ($p < 0.05$*).

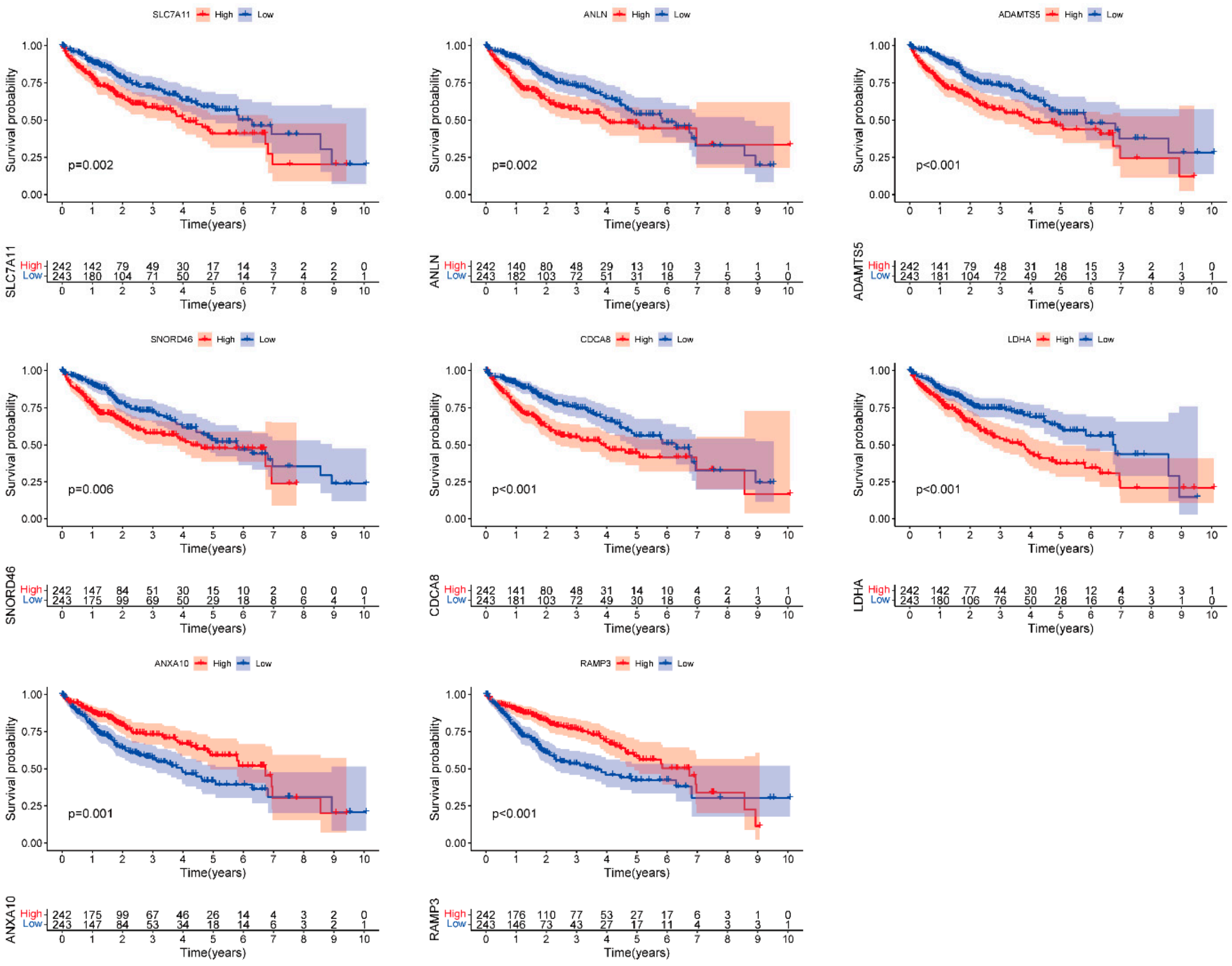

Figure S3. Kaplan–Meier overall survival curves showing statistically significant differences in prognosis based on the expression of the eight prognostic genes.

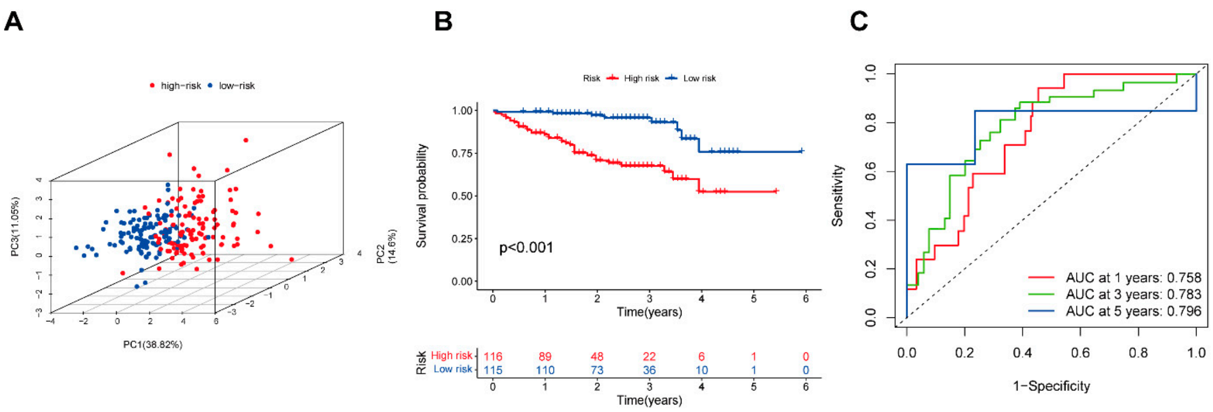

Figure S4. Validation of the m^{6}A-related prognostic model in the ICGC testing cohort

(A) 3D PCA plot illustrating clear discrimination between the high-risk and low-risk groups. (B) Kaplan–Meier overall survival analysis indicating that the high-risk group (n = 116) had significantly worse survival outcomes compared to the low-risk group (n = 115) (***p < 0.001). (C) Time-dependent ROC analysis demonstrating the robust predictive performance of the risk score, with AUC values greater than 0.75.

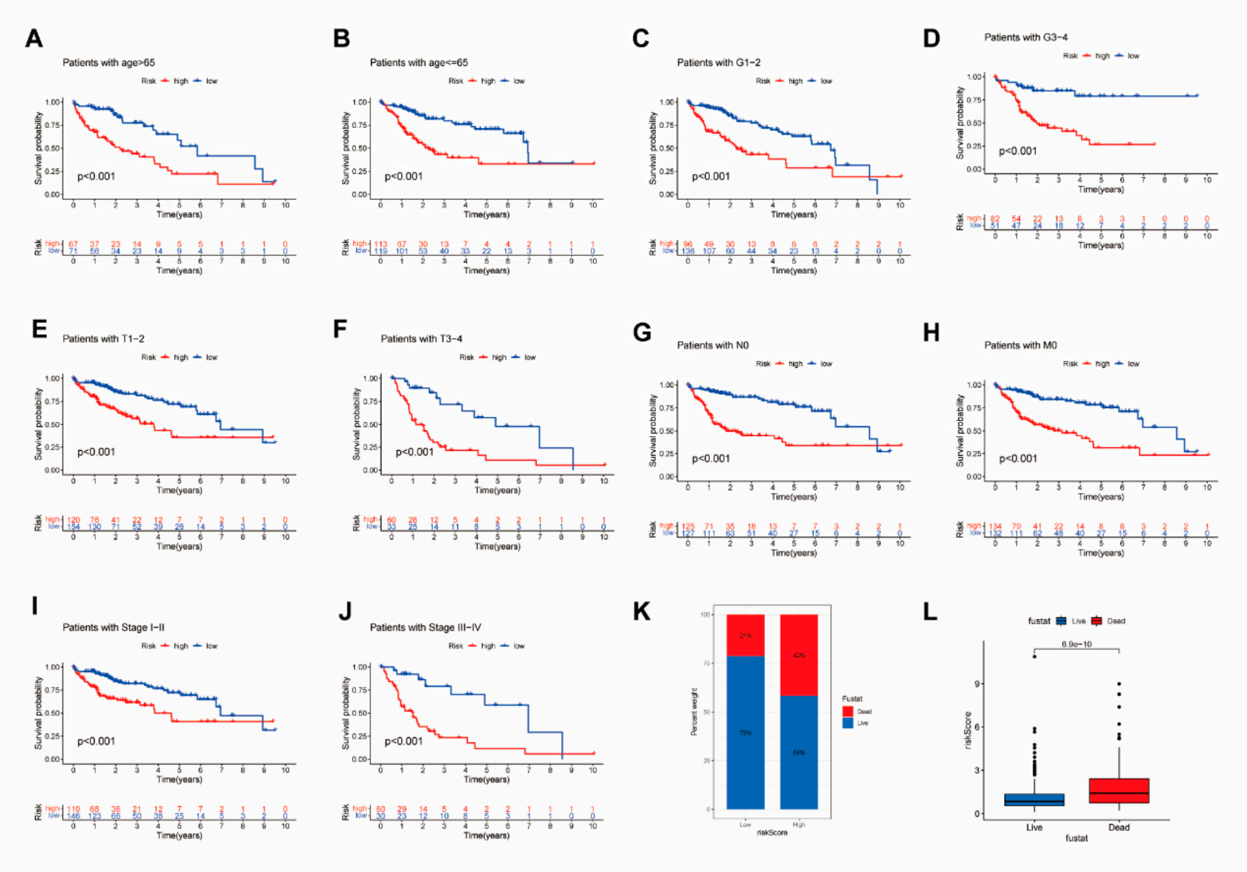

Figure S5. Kaplan–Meier survival curves of the eight-gene signature in the training cohort stratified by different clinical characteristics

(A, B) Age; (C) Tumor grade G1–G2; (D) Tumor grade G3–G4; (E) Tumor stage T1–T2; (F) Tumor stage T3–T4; (G) N0 stage (no regional lymph node metastasis); (H) M0 stage (no distant metastasis); (I) Clinical stage I–II; (J) Clinical stage III–IV. (K, L) Correlations between risk scores and clinical status.

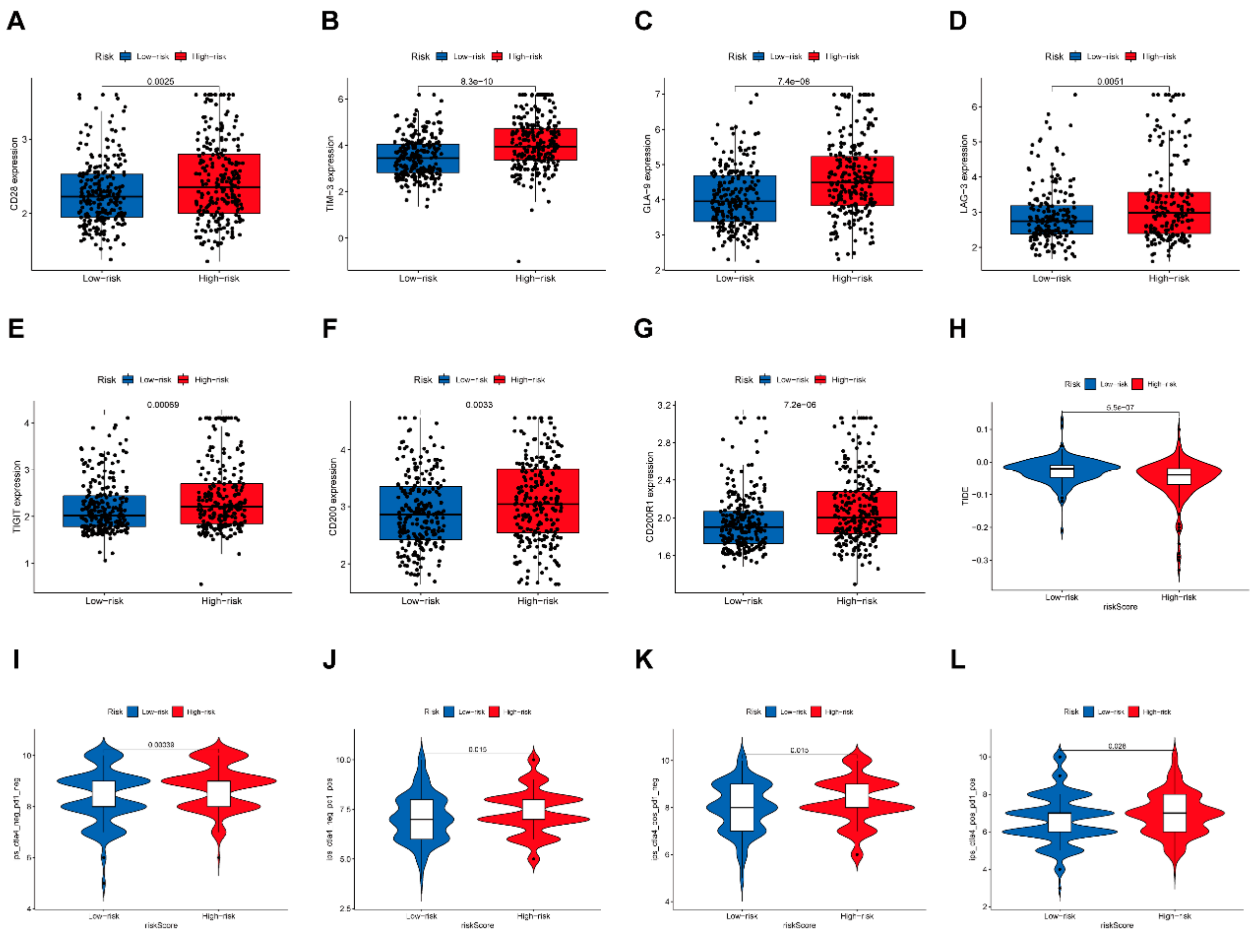

Figure S6. Associations between risk scores, immune checkpoint expression, and therapeutic sensitivity

(A–G) Expression levels of immune checkpoint genes (CD28, TIM-3, GLA-9, LAG-3, TIGIT, CD200, and CD200R1) in high- and low-risk groups (***p < 0.001). (H) Comparison of Tumor Immune Dysfunction and Exclusion (TIDE) scores between the high- and low-risk groups. (I–L) Immunophenoscore (IPS) analysis in different risk groups, reflecting tumor immunogenicity and predicted responsiveness to immunotherapy.

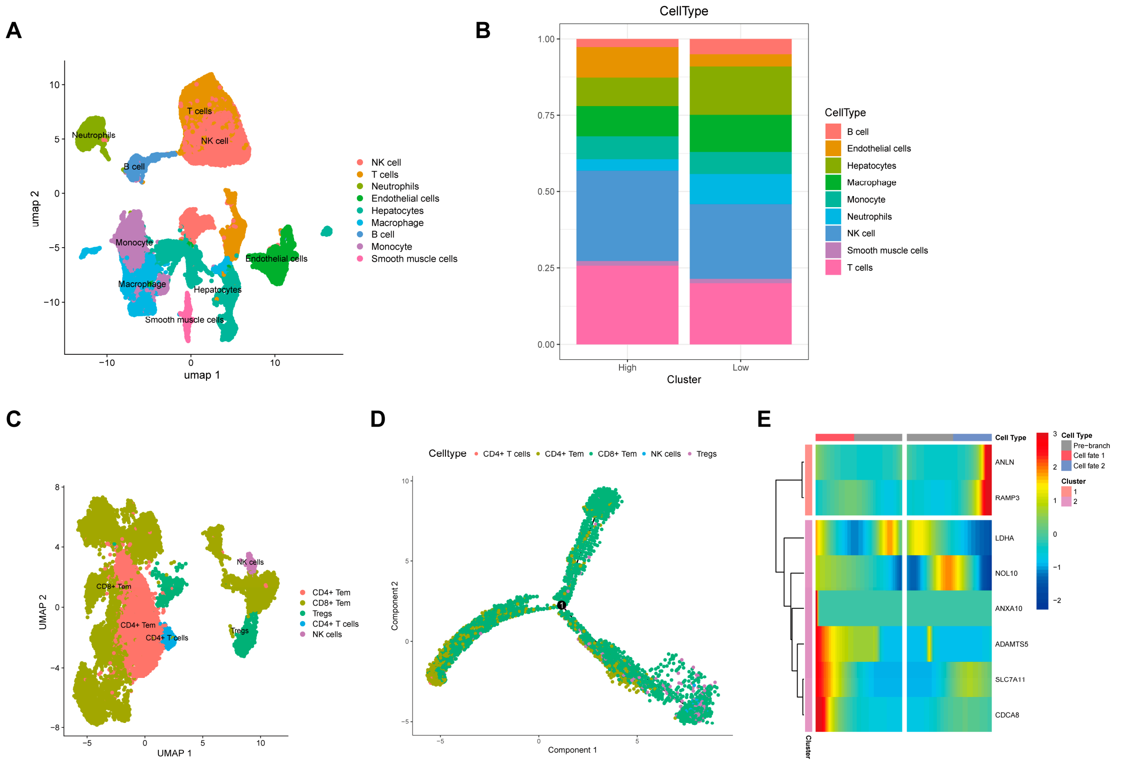

Figure S7. Differences in immune cell composition between risk groups and the association of prognostic genes with T cell differentiation.

(A) Based on the GSE202642 dataset, 7 HCC samples were analyzed, and 9 distinct cell types were identified according to marker gene expression. (B) Comparison of the proportions of different cell subsets between the low-risk and high-risk groups. (C) Annotation of T cell subtypes within clusters identified by UMAP. (D) Independent visualiza-tion of T cell subtypes at different stages along a pseudo-time trajectory, showing differentiation from pre-branch 1 (naive T cells) toward branch 1 (mature T cells), leading to two distinct fates. (E) Branch 1 further divided into two fate trajectories based on m6A-related gene expression along the pseudo-time axis.

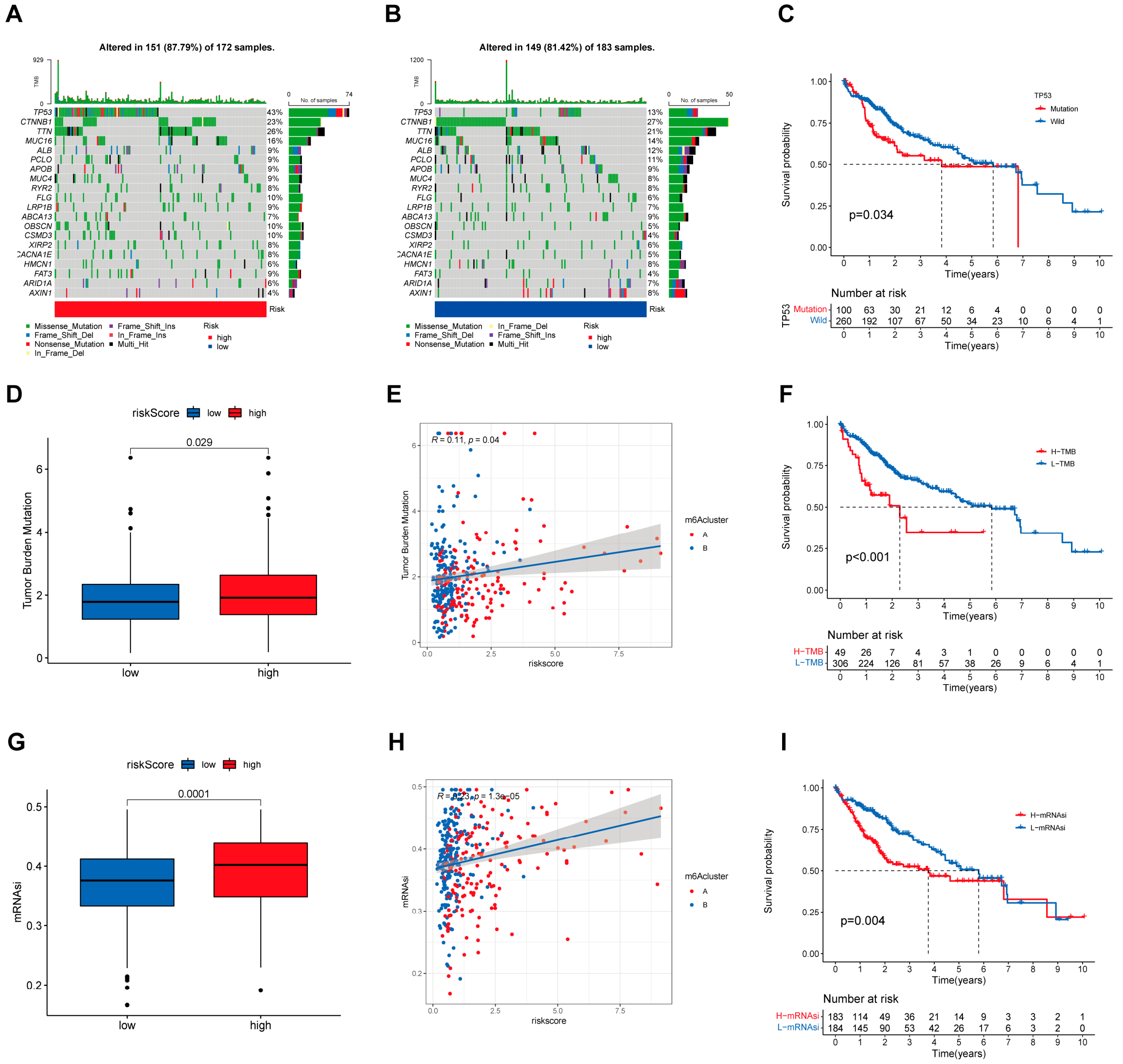

Figure S8. Comprehensive analysis of the risk score in HCC patients.

(A, B) Oncoplots illustrating the gene mutation patterns in the high-risk and low-risk groups. (C) Kaplan–Meier overall survival curves comparing TP53-mutant and TP53-wildtype HCC patients. (D) Boxplot comparing tumor mutation burden (TMB) between the high-risk and low-risk groups. (E) Correlation analysis showing a positive association between TMB and risk score. (F) Kaplan–Meier overall survival curves showing significantly poorer prognosis in HCC patients with high TMB compared to those with low TMB. (G) Boxplot comparing the mRNA stemness index (mRNAsi) between the high-risk and low-risk groups. (H) Correlation analysis revealing a positive correlation between mRNAsi and risk score. (I) Kaplan–Meier overall survival curves comparing HCC patients with high and low mRNAsi.

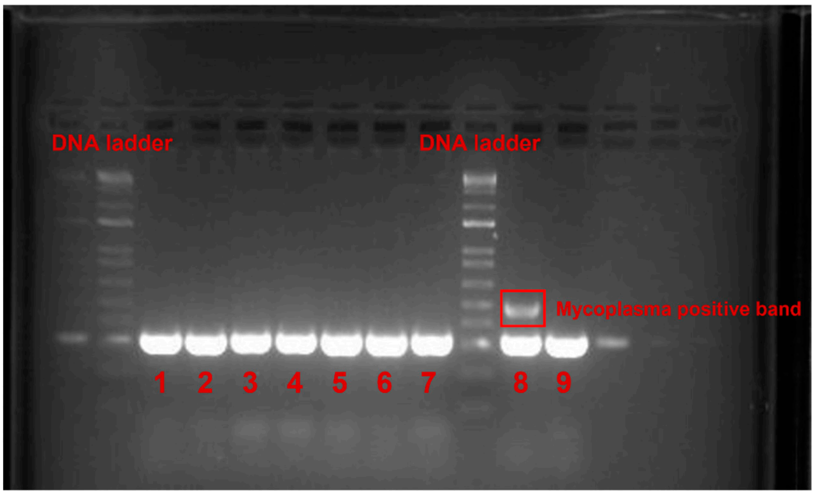

Legend:

1. HepG2
2. Hep3B
3. Huh7
4. HCCLM3
5. MHCC97H
6. SK-Hep-1
7. LO2
8. Positive control
9. Negative control

Figure S9. Mycoplasma PCR results for seven cell lines.

The presence of two bands indicates mycoplasma contamination.

Western blot 4B

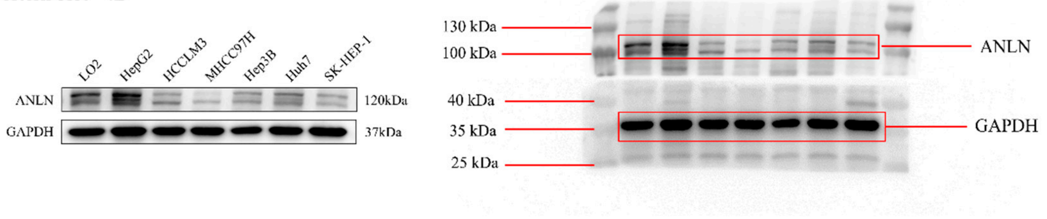

Western blot 4D

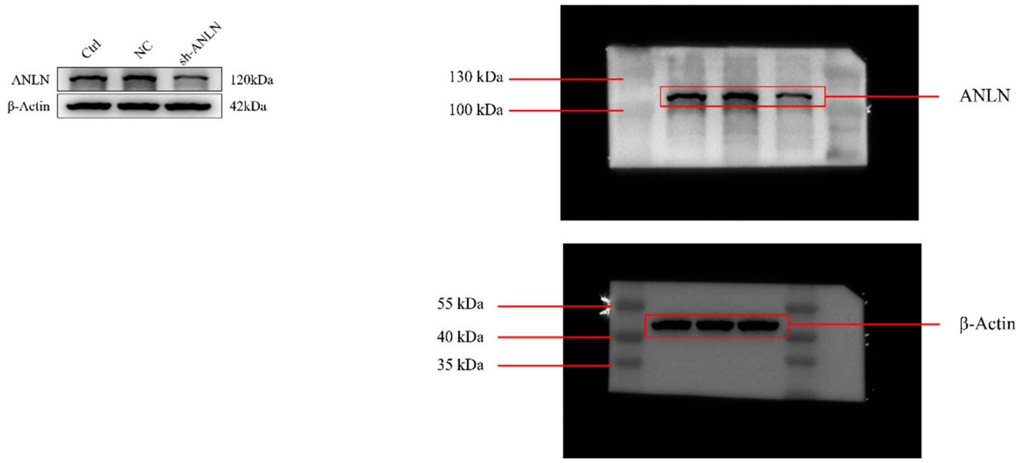

Figure S10. The original Western blot images. The blots marked by the red rectangle are the target proteins.
