# Supplementary material for: Stratification of Hepatocellular Carcinoma Using N6-Methyladenosine
Source: Cancers (Basel). 2025 Jul 2;17(13):2220. doi: 10.3390/cancers17132220 (PMC12249396; doi:10.3390/cancers17132220)
Supplement: Supplementary file 1 [file cancers-17-02220-s001.zip › cancers-3699019-supplementary/Table S1.pdf]

Table S1 The shRNA sequences and primer sequences used in this study

anillin-Homo-328	5′-GGTGGTGAAGAGAAATCTTGT-3′
anillin-Homo-934	5′-GCTACATTCTGTTCCCAAAGG-3′
anillin-Homo-1926	5′-GGATCAAGCATTAGCAGAAAG-3′
ANLN forward primer	ATCTTGCTGCAACTATTTGCTCC
ANLN reverse primer	TCCTGCTTAACACTGCTGCTA
